# *Candida* spp. in Denture Stomatitis: Prevalence, Microbial Load, and Antifungal Resistance Across Severity Levels

**DOI:** 10.3390/microorganisms13092057

**Published:** 2025-09-04

**Authors:** Marco Aurelio Fifolato, Lorena Mosconi Clemente, Adriana Barbosa Ribeiro, Viviane de Cássia Oliveira, Helio Cesar Salgado, Evandro Watanabe, Cláudia Helena Lovato da Silva

**Affiliations:** 1 Department of Dental Materials and Prosthesis, Ribeirão Preto School of Dentistry, University of São Paulo, Avenida do Café S/N, Ribeirão Preto 14040-904, São Paulo, Brazil; marcofifolato@usp.br (M.A.F.); lorena.clemente@usp.br (L.M.C.); driribeiro@usp.br (A.B.R.); vivianecassia@usp.br (V.d.C.O.); 2 Centro de Investigación Biomédica en Red de Enfermedades Respiratorias (Ciberes), Hospital Clinic de Barcelona, Villaroel, 170, 08036 Barcelona, Spain; 3 Department of Physiology, Ribeirão Preto Medical School. University of São Paulo, Avenida do Café S/N, Ribeirão Preto 14040-904, São Paulo, Brazil; hcsalgado@fmrp.usp.br; 4 Department of Restorative Dentistry, Ribeirão Preto School of Dentistry, University of São Paulo, Avenida do Café S/N, Ribeirão Preto 14040-904, São Paulo, Brazil; ewatabane@forp.usp.br

**Keywords:** complete denture, *Candida* spp., denture stomatitis, susceptibility, antifungals

## Abstract

Complete dentures (CD) are prone to biofilm formation, particularly by Candida species, which may lead to denture stomatitis (DS). As edentulism remains highly prevalent among the global ageing population, denture-related infections represent a significant public health concern. The novelty of this study lies in integrating the clinical severity of DS with the prevalence, microbial load, and antifungal susceptibility profile of *Candida* spp., providing new insights into the pathogenesis and therapeutic management of this condition. Biofilm from the CD and palate was seeded for prevalence and microbial load. The identification of strains was confirmed molecularly, and susceptibility to micafungin, nystatin, fluconazole, and miconazole was assessed by the broth microdilution method. Prevalence was shown in percentage, microbial load was analyzed using a generalized linear model test, and susceptibility was assessed using Pearson’s Chi-square test (*p* < 0.05). *Candida albicans* was the most prevalent regardless of DS. However, a higher microbial load of *C. albicans* was observed with increased severity of DS (*p* = 0.038). Except for *Candida tropicalis*, the microbial load of the CD was higher than that of the palate. *C. tropicalis* showed resistance to fluconazole with increased severity of DS (*p* = 0.004). All strains were susceptible to nystatin and miconazole, and three were resistant to micafungin. The findings suggest that the prevalence of *Candida* spp. is not a determining factor in the variation in DS severity. Nevertheless, patients with severe inflammation harbor an increased load of *C. albicans* on both sites. Nystatin and miconazole appear to be effective treatments for DS.

## 1. Introduction

Despite significant advances in preventive dentistry, edentulism remains a public health challenge, particularly among individuals aged 65 years and older in many countries [1,2,3]. Rehabilitation of the stomatognathic system in these patients is often achieved using removable dentures made of acrylic resin [4]. However, the inherent properties of this material promote biofilm formation, which is characterized by a complex community of fungi and bacteria embedded in an extracellular matrix [5,6,7]. Under healthy conditions, microorganisms within the biofilm interact in a commensal manner. Yet, in response to immunological or environmental changes in the host, local infections such as denture stomatitis (DS) may develop [8,9,10,11].

DS is a chronic inflammation with a multifactorial etiology associated with poorly fitting dentures, continuous denture wear, hyposalivation, and, most importantly, inadequate hygiene. Poor hygiene is one of the most critical factors in the progression of inflammation due to the prevalence of *Candida* spp. [12,13,14,15,16,17]. Although commonly present as components of the human oral microbiota [18], this microorganism can act as an opportunistic pathogen in the presence of complete dentures, particularly when associated with biofilm [19].

Treatment of DS may include conservative methods, such as the implementation of hygiene protocols [20,21,22], and/or the prescription of antifungal agents [23,24]. The prescription of antifungal agents aims to treat inflammation immediately, and it should follow the recommended dosage and duration. The selection of an antifungal for treating *Candida* spp. infections should be based on their chemical properties, antifungal action, and the severity of the infection.

**Figure 1:**
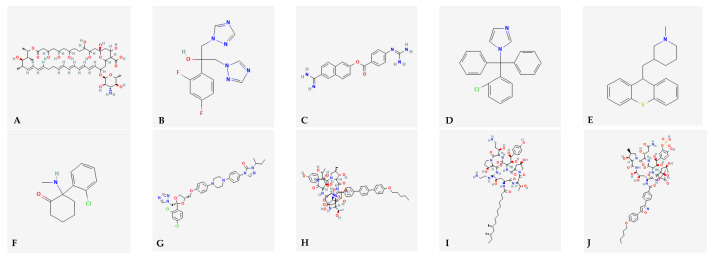
Chemical structures of the main antifungal agents evaluated: (**A**) nystatin; (**B**) Amphotericin B; (**C**) fluconazole; (**D**) clotrimazole; (**E**) miconazole; (**F**) ketoconazole; (**G**) itraconazole; (**H**) caspofungin; (**I**) anidulafungin; and (**J**) micafungin (https://pubchem.ncbi.nlm.nih.gov; accessed on 20 August 2025).

Among the antifungals used to treat DS, it is possible to list the polyenes, azoles, and echinocandins. Polyenes interact with ergosterol in fungal cell membranes, altering their permeability and integrity by forming transmembrane pores. This class includes nystatin and Amphotericin B, with the latter being widely used in cases of more severe infections. The azoles act in a fungistatic manner by inhibiting lanosterol 14-α-demethylase, encoded by the ERG11 gene, thereby inhibiting fungal ergosterol biosynthesis. Fluconazole, clotrimazole, miconazole, ketoconazole, and itraconazole are representatives of this class and are used to treat various types of fungal infections. The echinocandins class, represented by the antifungals caspofungin, anidulafungin, and micafungin, addresses the β-1,3-glucan synthesis in fungal cell walls. Echinocandins, which are frequently used in the treatment of systemic *Candida* infections, could potentially be considered for treating DS if the first-line treatments, with polyenes or azoles, prove ineffective [10,25,26] (Figure 1).

Relapse of DS is common after discontinuing treatment, creating a need for repeated interventions, which can lead to fungal resistance [27,28]. Besides drug resistance, virulence factors such as adhesion, biofilm formation, and the secretion of hydrolytic enzymes facilitate the persistence of fungi in host tissue and contribute to antifungal resistance [10,26,29]. However, it remains unclear whether antifungal resistance is associated with the profile of patients with DS or if similar resistance can also be observed in healthy individuals in the context of this inflammation [10,26,27,28,30,31,32,33].

Although the preferred treatment for DS prioritizes biofilm control and maintenance of denture hygiene, understanding the susceptibility of *Candida* spp. is essential in cases where conservative measures are insufficient. Such knowledge contributes to a broader understanding of yeast epidemiology and drug resistance, supporting the judicious and restricted use of antifungal agents, ensuring they are prescribed only when necessary.

Therefore, this study aimed to analyze the prevalence, microbial load, and antifungal susceptibility of *Candida* species in completely edentulous individuals, with varying severity levels of DS.

## 2. Materials and Methods

### 2.1. Selection of Individuals

This observational cross-sectional clinical study was conducted in accordance with the Declaration of Helsinki and was approved by the local Research Ethics Committee (CAAE 93712418.1.0000.5419). Participants were recruited between August 2021 and October 2023 and signed an informed consent form prior to their inclusion in the study.

Inclusion criteria were as follows: individuals of both sexes, in good general health, completely edentulous in both arches, and users of upper or bimaxillary complete dentures in clinically acceptable condition, fabricated with heat-polymerized acrylic resin and in use for at least 12 months.

Exclusion criteria included the following: individuals with systemic immunosuppression, history of cancer, autoimmune diseases, Chagas disease, use of nasogastric tubes (which may significantly alter oral microbiota), or uncontrolled chronic conditions such as diabetes mellitus. Individuals presenting with residual roots or dental implants were excluded because of potential differences in microbial profiles compared to fully edentulous individuals. One patient reporting a mental health condition was excluded due to difficulty in accurately responding to the questionnaire and data collection. Patients wearing removable partial dentures or dentures with visible repairs were also excluded, as these prostheses are made with different materials (e.g., metal frameworks or self-cured acrylic resin), which have distinct surface properties and may favor greater biofilm accumulation compared to heat-cured acrylic resin.

**Figure 2:**
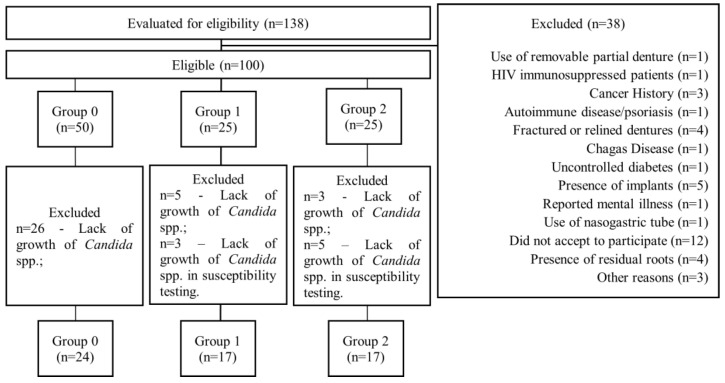
Flowchart of participant selection and grouping, following the STROBE guidelines.

Participants were clinically examined and stratified according to the presence or absence of denture stomatitis (DS), and only those who met all inclusion criteria were enrolled. The detailed screening and eligibility process is presented in the flowchart in Figure 2 of the Results section.

### 2.2. Denture Stomatitis Diagnosis

A researcher (L.M.C.) examined the participants’ palates, and the presence of DS was characterized according to the modified Newton classification [14]. Subsequently, a new grouping was proposed based on the inflammation score: Group 0 (G0)—score 0 for the absence of DS; Group 1 (G1)—score 1 (moderate inflammation) for Types IA and IB; and Group 2 (G2)—score 2 (severe inflammation) for Types II and III. Standardized photographs of the palate were then taken by the researcher (A.B.R.), focusing on the median raphe region [20] (Canon EOS Digital Camera, Canon EF Macro Lens 100 mm/2.8, and Canon ML3 Ring Flash, Canon Inc., Tokyo, Japan) to confirm the inflammation scores later. The confirmation was performed by an experienced masked researcher (C.H.S-L). Both C.H.S-L and L.M.C. were previously calibrated, achieving a Kappa value of 0.878.

For sample characterization, sociodemographic information regarding sex, age, marital status, education, living conditions, and income was collected by the researchers (M.A.F., L.M.C., and A.B.R.) using a standardized questionnaire developed by the researchers (A.B.R. and C.H.L-S).

### 2.3. Prevalence and Microbial Load of Candida *spp.* and Nakaseomyces Glabratus (Former Name: Candida glabrata)

Biofilm sampling was collected from the internal surface of the participants’ upper dentures and palates. A researcher (A.B.R.) removed the complete dentures, stored them in a sterile bag, and transferred the dentures to the laboratory. Then, a cytological sterile brush was rubbed on the palatal regions, and the active tip was stored in a sterile tube containing 1.5 mL of phosphate-buffered saline (PBS) [20,21,22].

In the laboratory, a masked researcher (V.C.O.) collected the biofilm from the upper denture in an aseptic area using the dissolution technique with a sterilized soft-bristle brush (Johnson & Johnson, São José dos Campos, SP, Brazil) and 10 mL of PBS. The resulting suspension was collected and transferred to an assay tube containing glass beads [20,21,22].

The suspensions from both the dentures and the palate were vortexed for 1 min (Phoenix, Araraquara, SP, Brazil), and 10-fold serial dilutions were subsequently prepared. The dilutions were seeded onto Petri dishes containing CHROMagar™ *Candida* (CHM), a specific culture medium for the isolation and identification of *Candida* spp. The Petri dishes were incubated (De Leo, Porto Alegre, RS, Brazil) at 35 °C for 48 h.

After the incubation period, the number of colonies was registered and expressed as Colony Forming Units per milliliter (CFU/mL) to quantify the microbial load. The identification of species was carried out based on color differentiation (researcher M.A.F.) [20,21,22] according to the manufacturer’s guidelines: light green, medium green, and dark green colonies were classified as *Candida albicans* complex (*Candida albicans* and *Candida dubliniensis*), purple or blue colonies as *Candida tropicalis*, and light purple or lilac colonies as *Nakaseomyces glabratus*. The isolated strains were stored at −80 °C in glycerol stocks for subsequent molecular identity confirmation.

To obtain reliable results regarding prevalence, microbial load, and antifungal susceptibility, the molecular identification was performed to confirm the species identified by the presumptive method. The data for prevalence (individual percentage) and microbial load (CFU/mL) were presented according to the molecular identification.

The molecular identification was conducted through Polymerase Chain Reaction (PCR)/restriction fragment length polymorphism (RFLP) fingerprints, targeting either a part of the intergenic spacer 2 or the entire intergenic spacer (IGS2: *C. albicans* and IGS: no-albicans) of ribosomal DNA as described by Cornet et al. [33]. Briefly, the researchers (M.A.F. and V.C.O.) extracted and purified the yeast’s DNA with phenol/chloroform (Merck, Burlington, MA, USA). For amplification, a mixture containing 10 pmol of each primer [IGS: 5′-CGATCTGCTGAGATTAAG-3′ (forward) and 5′-CTTAATCTTTGAGACAAGC-3′ (reverse); IGS2: 5′-TTAACTACAGTTGATCGGAC-3′ (forward) and 5′—CTTAATCTTTGAGACAAGC-3′ (reverse)], 5 nmol of dNTP (Qiagen, Hilden, Germany), 1.5 U Taq polymerase (Sigma Aldrich, St Louis, MO, USA), 10% Taq polymerase buffer (Sigma Aldrich), and 100 ng of DNA, in a final volume of 25 μL, was subjected to cycling thermal (initial denaturation at 94 °C for 5 min, succeeded by 35 cycles of 94 °C for 30 s, 52 °C for 30 s, 72 °C for 1 min, and a final extension at 72 °C for 7 min). After amplification, the PCR products were treated with 1.5 U of NlaIII (BioLabs, Hitchin, UK) or AluI (Sigma Aldrich) enzymes. The restriction fragments were separated on 2% agarose gel and observed under UV light. The fingerprint patterns were compared with those of commercial strains (*C. albicans* ATCC 10231; *C. tropicalis* ATCC 750; *Nakaseomyces glabratus* ATCC 2001).

### 2.4. Antifungal Susceptibility Analysis

Antifungal susceptibility was evaluated using the broth microdilution method, following the guidelines of the Clinical and Laboratory Standards Institute (CLSI M27-A4) [34]. The antifungal agents tested were micafungin, fluconazole, nystatin, and miconazole (all from Sigma Aldrich, St Louis, MO, USA). Stock solutions were prepared at 1280 µg/mL, according to the solubility properties of each compound: fluconazole and micafungin were dissolved in sterile distilled water, while nystatin and miconazole were dissolved in 100% dimethyl sulfoxide (DMSO). Aliquots were stored at −80 °C, protected from light.

Before testing, serial dilutions were prepared in RPMI-1640 medium (Sigma Aldrich), buffered with 3-(N-Morpholino) propanesulfonic acid (MOPS) at pH 7.0 and supplemented with 2% glucose, to achieve final concentrations ranging from 0.03 µg/mL to 16 µg/mL. *Candida* spp. were grown overnight, and cultures were adjusted to a final concentration of 1–5 × 10^3^ cells/mL using a Neubauer chamber (Precicolor; HBG Henneberg-Sander, Gießen, Germany). The test was performed in duplicate in 96-well microplates with 10 two-fold serial dilutions of each antifungal.

Two control conditions were included in each plate: positive control wells, containing *Candida* spp. inoculum without antifungal, to confirm fungal viability and growth; negative control wells, containing only medium without inoculum or antifungal, to verify sterility. The plates were incubated at 35 °C, with initial readings performed at 24 h and confirmation at 48 h, if required, for all species and antifungal agents, in accordance with the CLSI M27-A4 guidelines, using a stereomicroscope.

Fluconazole susceptibility was interpreted in accordance with the CLSI M27-A4 guidelines, using the established clinical breakpoints for *Candida* species: susceptible (S) at MIC ≤ 8 µg/mL, susceptible dose-dependent/intermediate (SDD/I) at MICs between 16 and 32 µg/mL, and resistant (R) at MIC ≥ 64 µg/mL. For miconazole and nystatin, a semi-quantitative scoring system based on growth inhibition observed in a 96-well plate was adopted. Since no official CLSI or European Committee on Antimicrobial Susceptibility Testing (EUCAST) clinical breakpoints exist for miconazole and nystatin, a semi-quantitative scoring system was adopted based on a 96-well broth microdilution assay, aligned with the methodology described in CLSI M27-A4 for turbidity-based readings [34]. Specifically, it was used Score 0: no visible growth (≈100% inhibition); Score 1: moderate turbidity (≈50% inhibition); or Score 2: strong turbidity (≈0% inhibition). The MIC was defined as the lowest concentration yielding at least 50% growth inhibition (score ≤ 1), in line with standard interpretive procedures. The Categorization Criteria were based on the following scoring system: susceptible (S): score = 0 (0% growth); susceptible dose-dependent (SDD): score = 1 (~50% growth); or resistant (R): score = 2 (>50% growth). This approach is consistent with established practices often used in the absence of official breakpoints, such as previous studies evaluating miconazole activity against *Candida* biofilms [35], and standard broth microdilution interpretive frameworks [36].

### 2.5. Data Analysis

The sociodemographic characteristics of participants from groups 0, 1, and 2 were qualitatively described. Data were tested for normality (Shapiro–Wilk test) and homoscedasticity (Levene test) to determine the appropriate statistical tests. The prevalence of *Candida* species by site and denture stomatitis severity was presented as a percentage. The comparison of microbial load as a function of inflammation degree (groups 0, 1, and 2) and collection site (denture and palate) was performed for each *Candida* species using a generalized linear model with a post hoc Wald test. Microbial load was measured in CFU/mL and transformed into Log_10_(CFU+1). The antifungal susceptibility analysis of *Candida* spp. isolated from groups 0, 1, and 2 were performed using Pearson’s Chi-square Test. A significance level of 5% was adopted for all analyses, which were conducted using SPSS software (SPSS 21.0, Inc., Chicago, IL, USA).

## 3. Results

Of 100 participants assessed for eligibility via convenience sampling, which included 50 individuals without DS (50%) and 50 with DS (25% with DS score 1, and 25% with DS score 2), 26 from G0 were excluded due to the absence of *Candida* spp. growth. In the G1, 5 of the 25 eligible participants were excluded due to the absence of *Candida* spp. growth, and 3 were excluded because no *Candida* spp. growth occurred in the broth microdilution test. In the G2, 3 of the 25 eligible participants were excluded due to the absence of *Candida* spp. growth, and 5 were excluded because no *Candida* spp. growth occurred in the broth microdilution test (Figure 2).

### 3.1. Sociodemographic Data

The sociodemographic characteristics of participants in Groups 0 (no DS), 1 (mild/moderate DS), and 2 (moderate/severe DS) were found to be similar across groups. Most participants were female; aged between 60 and 75, with a mean age of 66.3 years; were married or living with family; and had completed elementary education. Most reported household incomes range from one to three minimum wages. These distributions were consistent across all three groups, indicating homogeneity in baseline sociodemographic variables. A detailed breakdown is provided in Table 1.

### 3.2. Prevalence and Microbial Load of Candida *spp.*

**Figure 3:**
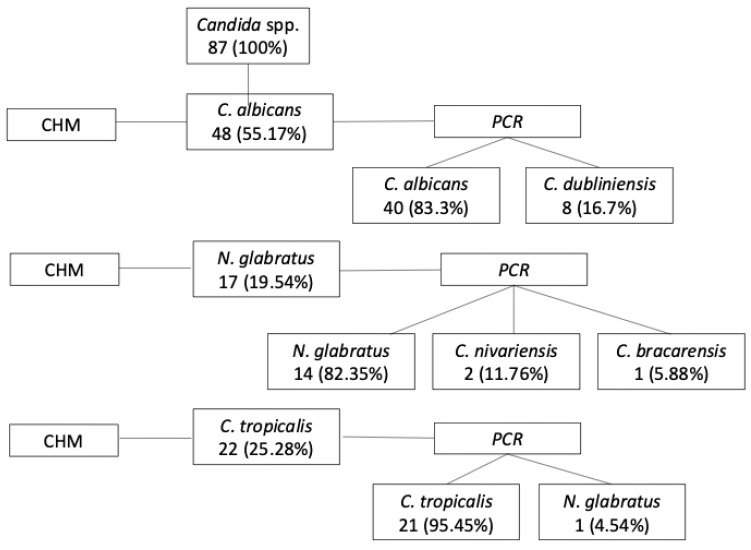
Number (%) of strains identified by presumptive method (CHM) and confirmed or not by PCR.

A total of 58 participants met all eligibility criteria and showed positive growth of *Candida* spp. in culture. From these individuals, 87 (100%) clinical isolates of *Candida* spp. were obtained, as some participants harbored more than one species. Based on presumptive identification using CHROMagar™ *Candida*, 48 isolates (55.17%) were identified as *Candida albicans*, 17 isolates (19.54%) as *N. glabratus*, and 22 isolates (25.28%) as *C. tropicalis*. Subsequent molecular identification confirmed that 40 (83.3%) of the 48 *C. albicans* isolates were correctly identified, while 8 (16.66%) were reclassified as *C. dubliniensis*. Among the *N. glabratus* isolates, 14 (82.35%) of 17 were confirmed, with 1 isolate (5.88%) identified as *C. bracarensis* and 2 isolates (11.76%) as *C. nivariensis*. Of the 22 *C. tropicalis* isolates, 21 (95.5%) were confirmed, and 1 (4.5%) was reclassified as *N. glabratus*. Overall, 75 (86.2%) out of 87 isolates were confirmed by molecular methods, indicating substantial agreement between phenotypic and genotypic identification, as illustrated in Figure 3 [37].

**Figure 4:**
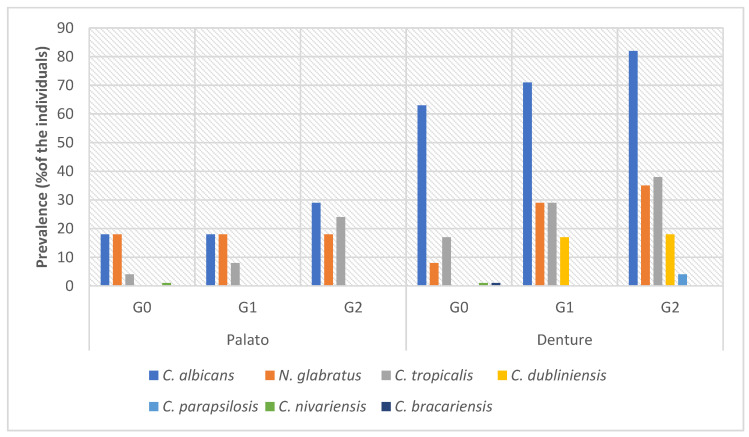
Prevalence (%) of the individuals with *Candida* spp. in accordance with site (palate and denture) and DS score (G0, G1, and G2).

Regarding the prevalence of the strains identified by collection site (denture and palate) and DS score, it was found that *C. albicans* was the most common species in both sites (palate and denture), with the denture being the most contaminated site. *C. albicans* and *C. tropicalis* were more prevalent in the palate of individuals with a DS score of 2, while these same species were more prevalent in the denture of individuals with a score of 0. *N. glabratus* was more prevalent in the dentures of a greater number of individuals with a DS score of 2. Additionally, *C. dubliniensis* was more prevalent in the dentures of the individuals with scores 0 and 1 of the inflammation (Figure 4).

The descriptive analyses of microbial load based on DS score and collection site are shown in Table 2.

For *C. albicans*, the microbial load was influenced by the degree of DS (*p* = 0.038) and the collection site. There was no interaction between the factors (*p* = 0.261). The G2 had a higher load of *C. albicans* compared to the G0, while the G1 had intermediate load values between the G0 and G2 groups. The denture showed a significantly higher load of *C. albicans* than the palate (Table 3).

For *N. glabratus*, the load was influenced by the collection site (*p* = 0.001), with the denture [1.13 (0.59–1.66)] showing a higher CFU count than the palate [0.35 (0.11–0.60)]. The DS score (*p* = 0.104) and the interaction of the site × DS score (*p* = 0.301) did not affect the microbial load. For the load of *C. tropicalis*, there was interaction between collection site × DS score factors (*p* = 0.026). Comparison of means indicated that for G0, the highest microbial load was identified in the denture. For G1 and G2, there was no difference between the sites (Table 4).

### 3.3. Antifungal Susceptibility Analysis of Candida *spp.* Samples

The results of antifungal susceptibility analysis showed that, in general, the resistance of the *Candida* spp. was low. Among *C. albicans*, *C. tropicalis*, *N. glabratus* e *C. dubliniensis*, only *C. tropicalis* showed resistance to fluconazole; the more severe the inflammation, the greater the number of resistant strains (Table 5). Despite low prevalence, *C. nivariensis* (2 strains) and *C. bracarensis* (1 strain) were susceptible to micafungin, nystatin, and miconazole, and were dose-dependent to fluconazole, respectively.

## 4. Discussion

This observational cross-sectional study was developed with denture users who attended the Ribeirão Preto School of Dentistry. The sociodemographic characteristics of the population were similar to those in other studies, with the majority of participants being female, living with their families, married, having completed middle school, and belonging to low-income households. Regarding denture stomatitis, the majority (58.6%) of participants presented the inflammation [15,17,20,22].

Accurate species identification is critical for interpreting antifungal susceptibility results. Therefore, although the main focus of this study was not to compare identification methods, we adopted a cautious diagnostic approach by performing initial presumptive identification using CHROMAgar *Candida* followed by molecular confirmation by PCR-RFLP. This was conducted to ensure the reliability of the susceptibility data. The observed 85.4% agreement between the two methods, classified as substantial according to Landis and Koch (1977) [38], deserves mention. This finding reinforces the clinical usefulness of CHROMAgar *Candida* for initial screening, while highlighting the role of molecular methods when precise identification is necessary for guiding treatment decisions.

*C. albicans* showed the highest prevalence across the three groups, followed by *C. tropicalis* and *N. glabratus* [37,39,40]; overall, the microbial load of *Candida* spp. was higher on dentures compared to the palate, consistent with other studies [14,21]. These results confirm that dentures are an important niche for biofilm development, and considering that biofilm is considered an important virulence factor, it underscores the need for effective strategies to prevent and control biofilm formation to mitigate associated health risks [31]. *C. albicans* was more prevalent than *N. glabratus* and *C. tropicalis*, independent of the presence of stomatitis. In recent years, several studies have demonstrated that *C. albicans* is considered the main cause of denture-related stomatitis. In this study, it was the more prevalent species; however, it is important to mention that non-*C. albicans* species, such as *N. glabratus* and *C. tropicalis,* were frequently identified [15,17,21,22]. According to Silva et al. (2012) [29], the increasing identification of non-*albicans* species can be attributed to advancements in molecular diagnostics and culture methods, which have improved the precision and reliability of species-level identification.

An intriguing finding was the identification of *C. nivariensis* and *C. bracarensis* in the group without DS (G0). These species are not frequently isolated but have been emerging over the last decade [41,42,43,44]. Phenotypically, they resemble *N. glabratus* [42], and chromogenic media and biochemical assays often fail to distinguish between these species because they share similar colony morphology and metabolic profiles.

Regarding antifungal susceptibility, a significant difference was observed for *C. tropicalis*, which showed an elevated level of resistance to fluconazole with increased severity of stomatitis. Overall, there was a high fluconazole resistance rate, especially among non-*albicans* strains. Oral fluconazole is effective for treating oral candidiasis unresponsive to topical treatment. However, suboptimal dosing may contribute to the development of resistance across *Candida* spp. [45]. For the species of *C. nivariensis* and *C. bacarensis*, all strains were susceptible to the antifungal micafungin, nystatin, and miconazole, and were dose-dependently susceptible to fluconazole. It should be noted that these results must be interpreted with caution, given the low prevalence of *C. nivariensis* (2 strains) and *C. bracarensis* (1 strain). Micafungin is a selective inhibitor of fungal 1,3-β-D-glucan synthesis and is commonly used for invasive and esophageal candidiasis [46] and is typically reserved for severe cases due to its intravenous administration. Nystatin and miconazole are commonly used to treat oral candidiase, and fluconazole is used when the infection does not respond to nystatin and miconazole use. According to the literature, the susceptibility of these species remains unclear, as variations in susceptibility have been observed across different studies [41,47,48].

Micafungin is typically reserved for severe cases due to its intravenous administration. The strains identified as *C. nivariensis* and *C. bracarensis* were either susceptible or susceptible dose-dependent to antifungal agents. According to the literature, the susceptibility of these species remains unclear, as variations in susceptibility have been observed across different studies [41,47,48].

Regarding fluconazole resistance rates, the resistance rates for *C. tropicalis* (up to 71.4% in group 1) and *N. glabratus* (up to 66.7%) appear higher than those commonly reported for oral isolates, which typically range from 10% to 30% for *C. tropicalis* and 20% to 50% for *N. glabratus*, depending on the population studied and prior antifungal exposure [49]. This discrepancy may reflect differences in geographic location, sampling methodology, or prior antifungal use in our patient cohort.

It is important to discuss the lack of cross-resistance between miconazole and fluconazole. Despite the observed fluconazole resistance, all isolates remained susceptible to miconazole (Table 5). This lack of cross-resistance can be explained by the distinct mechanisms of action and molecular targets of these antifungal agents. While fluconazole acts by inhibiting lanosterol 14α-demethylase (ERG11), impairing ergosterol synthesis, miconazole exhibits additional membrane-disruptive effects and generates reactive oxygen species (ROS), contributing to its fungicidal activity [50,51]. Moreover, azole efflux pumps that mediate fluconazole resistance may have less impact on the efficacy of miconazole [52].

A further important issue of this study is that *C. nivariensis* was less susceptible than *N. glabratus* to fluconazole, commonly used in the treatment of candidiasis. This result is in accordance with Borman et al. (2008) [53]. Figueiredo-Carvalho et al. (2016) [42] evaluated the susceptibility profile of *C. nivariensis*, confirming its resistance to azole drugs typically used for treating candidiasis. Consequently, special monitoring of the presence of *C. nivariensis* is required, especially for elderly and immunosuppressed patients [42,43,44].

An important aspect of our study relies on the fact that all isolates were susceptible to nystatin and miconazole. This finding is significant since nystatin, a topical and oral antifungal with activity against many yeasts, including *C. albicans*, is widely used for treating cutaneous and oropharyngeal candidiasis. Due to its lack of oral absorption, nystatin has not been associated with drug-induced liver injury [54]. Nystatin and miconazole are the most commonly used topical antifungals and are effective, though they require prolonged use to eradicate infections. Miconazole formulations are more patient-friendly; however, potential drug interactions should always be assessed before prescription [55].

Regarding the results, micafungin must be interpreted with care. According to Perlin (2015) [56], echinocandin resistance among *Candida* species is uncommon, except in the case of *N. glabratus,* and it typically develops during therapy. Echinocandin action induces a variety of cellular stress response pathways, creating drug-adapted persistent states that may ultimately break through and form FKS-resistant mutants. Among the host factors promoting resistance are biofilm formation, and in this study, *Candida* spp. organized in biofilm form were collected from the inner surfaces of the dentures. However, this study did not include a question for participants regarding their previous use of antifungal agents of the echinocandins class or perform confirmation through molecular methods. Then, this situation could be considered as a limitation of the study, and future studies could incorporate the molecular methods to confirm these results [56].

Another point that can be considered as a methodological limitation of this study concerns the glucose concentration used in the RPMI-1640 medium during antifungal susceptibility testing. Although the CLSI protocol (document M27-A4) recommends the use of 0.2% glucose, this study used commercially available RPMI-1640 with 2% glucose, as supplied by the manufacturer (Sigma-Aldrich). This formulation, although not in accordance with the CLSI recommendation, is compatible with protocols adopted by other international committees, such as EUCAST, and provides favorable conditions for the growth of *Candida* spp. in vitro. However, it is recognized that higher glucose concentrations can alter the activity of certain antifungals, especially echinocandins, by influencing the composition of the fungal cell wall and cellular stress mechanisms. This explanation is supported by evidence showing that elevated glucose levels can stimulate cell wall synthesis, particularly of β-1,3-glucan and chitin, thereby reducing the accessibility of echinocandins to their target enzyme (β-1,3-glucan synthase) [57]. Moreover, glucose-induced stress responses may activate compensatory signalling pathways that reinforce the cell wall and ultimately diminish antifungal susceptibility. Therefore, the results obtained should be interpreted with caution when compared directly with breakpoints established by CLSI. Future comparative analyses with different glucose concentrations may contribute to assessing the impact of this variation on the determination of antifungal susceptibility.

## 5. Conclusions

In conclusion, the presence or severity of denture stomatitis does not appear to be determined by the overall prevalence of *Candida* spp. However, analysis of the microbial load suggests that C. albicans is associated with the severity of the condition, whereas the burden of *C. tropicalis* and *N. glabratus* does not seem to play a significant role. Finally, both nystatin and miconazole remain effective therapeutic options for denture stomatitis, as all isolates were susceptible to these antifungal agents.

## Figures and Tables

**Table 1:** Sociodemographic characteristics of participants in groups 0, 1, and 2, according to the inflammation score.

Outcomes	Groups		
	0	1	2
Sex			
Female	19 (79.2%)	13 (76.5%)	13 (76.5%)
Male	5 (20.8%)	4 (23.5%)	4 (23.5%)
Marital status			
Single	3 (12.5%)	2 (11.7%)	0 (0.0%)
Married	8 (33.3%)	13 (76.5%)	9 (53.0%)
Divorced	7 (29.2%)	1 (5.9%)	4 (23.5%)
Separated	1 (4.2%)	0 (0.0%)	0 (0.0%)
Widowed	5 (20.8%)	1 (5.9%)	4 (23.5%)
Lives With			
Alone	4 (16.7%)	3 (17.6%)	1 (5.9%)
Friend	0 (0.0%)	1 (5.9%)	0 (0.0%)
Family	20 (83.3%)	13 (76.5%)	16 (94.1%)
Education			
Illiterate	1 (4.2%)	2 (11.8%)	0 (0.0%)
Elementary School	4 (16.7%)	4 (23.5%)	2 (11.8%)
Middle School	14 (58.3%)	9 (52.9%)	9 (53.0%)
High School	5 (20.8%)	1 (5.9%)	5 (29.3%)
College or Higher	0 (0.0%)	1 (5.9%)	1 (5.9%)
Income (Minimum salary) *			
1–3 salaries	22 (91.7%)	17 (100%)	17 (100.0%)
4–7 salaries	2 (8.3%)	0 (0.0%)	0 (0.0%)

* National minimum salary of BRL 1320.00 (https://www.gov.br, accessed on 10 July 2025).

**Table 2:** Descriptive analysis of the microbial load (CFU/mL+1) of *Candida* species on the palate and denture and DS score (Groups 0, 1, and 2).

		*C. albicans*		*N. glabratus*		*C. tropicalis*		*C. dubliniensis*		*C. nivariensis*		*C. bracarensis*	
Groups		Palate	Denture	Palate	Denture	Palate	Denture	Palate	Denture	Palate	Denture	Palate	Denture
**0**	Mean	0.23	2.19	0.14	0.45	0.17	1.58	0.00	0.77	0.13	0.09	0.00	0.14
	SD	0.62	2.05	0.57	1.60	0.61	2.25	0.00	1.86	0.00	0.00	0.00	0.00
	Median	0.00	2.25	0.00	0.00	0.00	0.00	0.00	0.00	0.00	0.00	0.00	0.00
	CI	(−0.08;0.44)	(1.25;2.98)	(−0.10;0.38)	(−0.20;1.15)	(−0.08;0.43)	(0.69;2.59)	0.00	(0.01;1.58)	0.00	0.00	0.00	0.00
**1**	Mean	0.3	3.13	0.4	1.28	0.61	1.15	0.00	0.79	0.00	0.00	0.00	0.00
	SD	0.71	3.10	1.00	2.25	1.47	2.01	0.00	1.86	0.00	0.00	0.00	0.00
	Median	0.00	2.60	0.00	0.00	0.00	0.00	0.00	0.00	0.00	0.00	0.00	0.00
	CI	(−0.05;0.68)	(1.76;4.44)	(−0.09;0.94)	(0.21;2.51)	(−0.12;1.40)	(0.18;2.25)	0.00	(−0.12;1.79)	0.00	0.00	0.00	0.00
**2**	Mean	0.61	3.71	0.48	1.48	0.69	0.9	0.00	0.00	0.00	0.00	0.00	0.00
	SD	1.06	2.31	1.16	2.27	1.41	1.79	0.00	0.00	0.00	0.00	0.00	0.00
	Median	0.00	2.31	0.00	0.00	0.00	0.00	0.00	0.00	0.00	0.00	0.00	0.00
	CI	(0.10;1.19)	(2.40;4.77)	(−0.09;1.11)	(0.40;2.73)	(0.00;1.46)	(0.03;1.87)	0.00	0.00	0.00	0.00	0.00	0.00

SD = Standard deviation. CI = Confidence interval.

**Table 3:** Microbial load (CFU Log_10_+1) of *C. albicans* according to inflammation groups and site.

		Median	IC	*p* *
Groups	0	1.14 ^a^	0.69–1.59	0.038
	1	1.70 ^ab^	1.05–2.36	
	2	2.11 ^b^	1.50–2.72	
Site	Denture	2.93	2.33–3.53	<0.001
	Palate	0.37	0.16–0.59	

* Generalized linear model with Wald post-test. CI = Confidence interval. Different letters (a and b) indicate statistical differences among groups.

**Table 4:** Microbial load (CFU Log_10_+1) of *C. tropicalis* according to inflammation groups and site.

		Groups			*p* *
		0	1	2	
Denture	Median	1.64 ^Aa^	1.22 ^Aa^	0.95 ^Aa^	0.026
	CI	0.69–2.59	0.18–2.25	0.03–1.87	
Palate	Median	0.18 ^Ab^	0.64 ^Aa^	0.73 ^Aa^	
	CI	−0.08–0.43	−0.12–1.40	0.00–1.46	

* Generalized linear model with Wald post-test. CI = Confidence interval. Capital letters (A) compare groups (DS) and lowercase letters (a and b) compare sites (denture × palate); different letters indicate statistical differences.

**Table 5:** Comparison of the strains susceptible (S), susceptible dose-dependently (SDD), or resistant (R) to antifungals for each inflammation group.

		Groups	S	SDD	R	p *
*C. albicans*	Micafungin	0	14 (100%)	0	0	** 0.325
		1	12 (92.3%)	0	1 (7.7%)	
		2	12 (100%)	0	0	
	Fluconazole	0	8 (57.1%)	0	6 (42.9%)	** 0.639
		1	8 (61.5%)	1 (7.7%)	4 (30.8%)	
		2	7 (58.3%)	0	5 (41.7%)	
	Nystatin	0	14 (100%)	0	0	#
		1	13 (100%)	0	0	
		2	12 (100%)	0	0	
	Miconazole	0	14 (100%)	0	0	#
		1	13 (100%)	0	0	
		2	12 (100%)	0	0	
*N. glabratus*	Micafungin	0	3 (100%)	0	0	** 0.379
		1	5 (83.3%)	0	1(16.7%)	
		2	6 (100%)	0	0	
	Fluconazole	0	1 (33.3%)	0	2(66.7%)	** 0.375
		1	2 33.3%)	0	4(66.7%)	
		2	1 (16.7%)	2 (33.3%)	3 (50%)	
	Nystatin	0	3 (100%)	0	0	#
		1	6 (100%)	0	0	
		2	6 (100%)	0	0	
	Miconazole	0	3 (100%)	0	0	#
		1	6 (100%)	0	0	
		2	6 (100%)	0	0	
*C. tropicalis*	Micafungin	0	9 (90%)	0	1 (10%)	** 0.463
		1	7 (100%)	0	0	
		2	4 (100%)	0	0	
	Fluconazole	0	8 (80%)	0	2 (20%)	*** 0.004
		1	0	2 (28.6%)	5 (71.4%)	
		2	2 (50%)	0	2 (50%)	
	Nystatin	0	10 (100%)	0	0	#
		1	7 (100%)	0	0	
		2	4 (100%)	0	0	
	Miconazole	0	10 (100%)	0	0	#
		1	7 (100%)	0	0	
		2	4 (100%)	0	0	
*C. dubliniensis*	Micafungin	0	4 (100%)	0	0	#
		1	2 (100%)	0	0	
		2	1 (100%)	0	0	
	Fluconazole	0	3 (75%)	0	1 (25%)	** 0.144
		1	2 (100%)	0	0	
		2	0	0	1 (100%)	
	Nystatin	0	4 (100%)	0	0	#
		1	2 (100%)	0	0	
		2	1 (100%)	0	0	
	Miconazole	0	4 (100%)	0	0	#
		1	2 (100%)	0	0	
		2	1 (100%)	0	0	

* Pearson qui-square test; ** no difference between susceptible and resistant strains; *** there was a difference between susceptible and resistant strains; # all strains were susceptible; S: susceptible; SDD: susceptible dose-dependent; R: resistant.

## Data Availability

The datasets presented in this article are not readily available because the data are part of an ongoing study. Requests to access the datasets should be directed to the corresponding author.
